# 1111. Lack of Effectiveness of Colistin in Carbapenem-Resistant *Acinetobacter baumannii* : A Retrospective Single Center Cohort Study

**DOI:** 10.1093/ofid/ofad500.084

**Published:** 2023-11-27

**Authors:** Si-Ho Kim, Cheon-Hoo Jeon, Yu Mi Wi

**Affiliations:** Division of Infectious Diseases, Samsung Changwon Hospital, Sungkyunkwan University, Changwon, Korea., Changwon, Kyongsang-namdo, Republic of Korea; Samsung Changwon Hospital, Changwon, Kyongsang-namdo, Republic of Korea; Samsung Changwon Hospital, Changwon, Kyongsang-namdo, Republic of Korea

## Abstract

**Background:**

*Acinetobacter baumannii* is a major pathogen for nosocomial infection. The emerging resistance of *A. baumannii* to carbapenem limits the appropriate antimicrobial agent options. Colistin has been used for the treatment of carbapenem-resistant *A. buamannii* (CRAB), but there were only a few studies evaluating effectiveness of colistin with appropriate controls. In this regard, we investigated the effectiveness of colistin in treating pneumonia caused by CRAB compared to CRAB pneumonia treated with no active drug.

**Methods:**

Adult patients (≥ 18 years old) with CRAB isolated from their respiratory specimens were screened. Then, patients with pneumonia who were treated with colistin monotherapy (colistin group) as active antibiotics based on antimicrobial susceptibility and without any active antibiotics (no active drug group) were included. Pneumonia is defined as a new infiltrate on chest image and two of the following: 1) hyper or hypothermia (≥ 37.8 or ≤ 36.0 °C), 2) purulent respiratory secretion, 3) leukocytosis, and 4) hypoxemia. The primary outcome was 30 days of all-cause mortality. The secondary outcome was acute kidney injury within 30 days after pneumonia diagnosis or colistin administration. Only the patients who had baseline creatinine less than 2mg/dL were evaluated for the secondary outcome.

**Results:**

Among 828 patients with CRAB in their respiratory specimens, 45 patients were treated with colistin, and 123 received no active antibiotics susceptible to CRAB. Most of the CRAB pneumonia was hospital-acquired pneumonia (91.1%), and 51.1% of patients were ventilator-associated pneumonia. Purulent respiratory secretion (100% vs. 87.8%, P=0.012) and use of carbapenems (71.1% vs. 48.8%, P=0.010) were more frequent in the colistin group. There was no difference in 28 days of all-cause mortality in colistin (41.7%) and no active drug group (43.9%) in raw (P=0.62) and adjusted analysis (adjusted HR 0.72, 95% CI 0.45-1.2). Incidence of acute kidney injury was numerically common in the colistin group (65.3% vs. 39.0% ) but no statistical significance was observed (P=0.143).

Survival curve since the onset of CARB pneumonia

**Figure:**
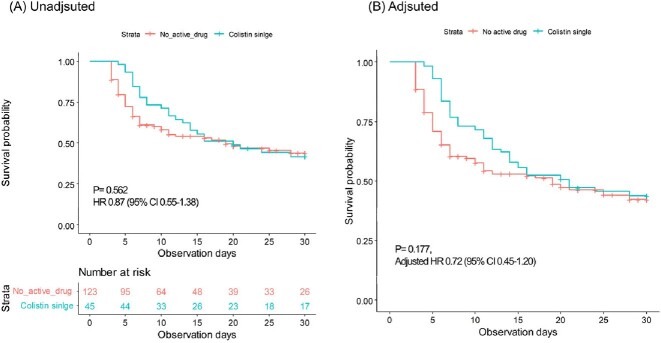

**Conclusion:**

Colistin might not have survival gain for the treatment of CRAB pneumonia. Newer effective antimicrobials effective for CRAB should be developed, evaluated, and rapidly applied to clinical practice.

**Disclosures:**

**All Authors**: No reported disclosures

